# Activating the souls of the older adults: insights into the psychological well-being of older adults through social interactions in the Guangdong-Hong Kong-Macao Greater Bay Area

**DOI:** 10.3389/fpubh.2025.1461481

**Published:** 2025-03-05

**Authors:** Xianglei Zhu, Qian Du, Yufen Li, Yucheng Zhu, Chengwei Ge, Jiawen Chen

**Affiliations:** ^1^School of Humanities and Management, Guangdong Medical University, Dongguan, China; ^2^School of Humanities, Taiwan National Chi Nan University, Taiwan, China; ^3^Qu Qiubai School of Government, Changzhou University, Jiangsu, China; ^4^School of Health Care Security, Shandong First Medical University, Jinan, China; ^5^The Affiliated Dongguan Songshan Lake Central Hospital, Guangdong Medical University, Dongguan, China

**Keywords:** older adults, mental health, social interaction, intergenerational support, Guangdong-Hong Kong-Macao Greater Bay Area

## Abstract

**Background/objectives:**

Addressing the challenges faced by the older adults in the Guangdong-Hong Kong-Macao Greater Bay Area (GHMGBA) in terms of mental health and enhancing their well-being is pivotal for achieving the goals of cultural integration and intercity connectivity.

**Methods:**

This study, grounded in social exchange theory, conducted an analysis using survey data collected from 6,500 older adults individuals in the Greater Bay Area (GBA). By constructing regression models, the research explores the impact of social interaction on the mental health of older adults individuals in the GBA.

**Results:**

The findings reveal that any form of social interaction among the older adults in the GBA significantly improves their mental health. However, the promotional effects vary significantly across different regions. In comparison to Guangdong, the impact of social interaction on the mental health of older adults individuals is more pronounced in Macao and Hong Kong. Notably, there is no significant difference in the impact of older adults social interaction on mental health between Hong Kong and Macao. Furthermore, the study identifies that intergenerational support plays an intermediary role in the social interactions and mental health of older adults individuals in the GBA.

**Conclusion:**

In the GBA should strengthen support for the social interaction of the older adults, promote communication among family members, and increase opportunities for the older adults to engage in social interactions. Future aging policies in the GBA should actively encourage downward intergenerational support, and intensify the exchange of intergenerational support.

## 1 Introduction

As a result of declining fertility and increasing longevity, the proportion and size of older populations are increasing in countries around the world (1). China has a large older adults population, with 267 million people aged 60 and above by the end of 2021, accounting for 18.9% of the total population. As a result, China is facing many problems of an aging population. First of all, a series of problems such as aging of the older adults organism and deteriorating health tend to trigger psychological depression. Secondly, the loss of self-worth and the reduction of social relationships and emotional support after retirement also tend to trigger psychological problems. Currently, more than 20% of Chinese older adults people face psychological depression, which brings a heavy burden to families and society. It can be seen that the importance of older adults’ mental health (MH) to the issue of population aging. Enhancing the MH of older adults is beneficial to extending their lifespan, improving quality of life and satisfaction, enhancing health equity, and achieving healthy aging and active aging. Therefore, it is necessary to investigate the factors influencing the MH of older adults and provide strategies to optimize their MH (2–5).

Recently, some studies have explored the MH of older adults at different levels. For example, He (6) and Chen (7) investigated the impact of older individuals’ social interaction habits on their MH. It has been discovered that the impact of various forms of social interaction on older individuals’ MH varies. Yuan (8) and Jiang (9) investigated the effects of older adults’ residence patterns on their MH and discovered that older adults’ MH was greatly improved when they lived with their children. Additionally, other researchers have investigated the effects of these characteristics on the MH of older individuals by starting with factors including Internet use (10, 11), urban and rural community surroundings (12), community aging services (2, 13), and social capital (1, 14).

However, there are still some limitations to existing studies. Most studies focus on exploring the correlation between social interaction and psychological well-being among older adults individuals in specific regions. There has yet to be research on the mechanisms through which social interaction affects the MH of the older adults in the Guangdong-Hong Kong-Macao Greater Bay Area (GHMGBA). Since the formal establishment of the GBA in 2019, its outstanding performance in economic vitality, cultural integration, technological innovation, and intercity connectivity has made it a unique highlight among the world’s four major bay areas. With the booming economic development of the GBA, profound changes are occurring in its social structure, and the impact of social interaction on the MH of the older adults is directly related to their quality of life in this diverse and highly developed urban cluster. Considering the intercity connectivity and cultural integration in the GBA, social interaction among the older adults in different regions may exhibit unique characteristics. Therefore, in-depth research into these differences will contribute to providing more personalized and accurate MH services for the older adults. Additionally, with the advancement of technology, the diversity and complexity of social interaction among the older adults are increasing, highlighting the urgent need for in-depth research on the impact of social interaction on the MH of the older adults. By gaining a profound understanding of the correlation between social interaction and the MH of the older adults in the GBA, we can more effectively formulate people-centered policies and services, enhancing the happiness and life satisfaction of the older adults in this vibrant and opportunistic region.

Given that Social Exchange Theory (SET) emphasizes the reciprocal nature of human relationships, it offers a valuable framework for understanding how older adults engage in social interactions by weighing the costs and benefits. Social interactions, such as time spent in activities and emotional exchanges, represent “costs” for older adults, while the social support, recognition, and improved psychological well-being they gain are the “benefits” or rewards. SET helps to explain why and how older adults might choose to engage in certain social activities and how these choices affect their MH. Moreover, by applying SET, this study explores not only the direct effects of social interaction but also the mediating role of intergenerational support, as this exchange of resources (financial, emotional, etc.) plays a key role in determining the psychological outcomes of social relationships.

Therefore, based on the Social Exchange Theory, this study explores the impact of social interaction and intergenerational support among the older adults in the GHMGBA on MH. The research team conducted a questionnaire survey from February 2023 to September 2023, involving 6,500 individuals aged 65 and above in these three regions of the GBA. The study investigates the specific mechanisms through which social interaction among the older adults in different regions affects their MH. This research not only provides guidance for the older adults in the GBA to choose social activities that improve their health but also offers significant assistance in promoting the health and well-being of the older adults population in the GBA, fostering cultural integration and intercity connectivity, and contributing to the implementation of the national strategy for population aging.

## 2 Theoretical basis and research hypotheses

### 2.1 Social exchange theory

Social exchange theory asserts that all human activities and social relationships are driven by rewards and costs. Benefits and costs are the two major elements of the theory (15). Among them, benefits include intrinsic gains such as recognition, attention from others, and emotional satisfaction, as well as extrinsic rewards like material acquisitions or instrumental services (16). Costs can take the form of physical effort, time spent, or other resources invested. This study conceptualizes social interaction as the physical and time investment older adults make in various activities. Through these engagements, older adults can gain positive emotions such as pleasure, relaxation, and a sense of fulfillment. From the perspective of Social Exchange Theory (SET), the reciprocal nature of these interactions is critical: older adults invest their time and energy into social interactions (the “costs”), but in return they receive emotional support, companionship, and improved mental health (the “benefits”). The balance of these costs and benefits helps to explain not only why older adults choose to engage in social activities but also how these activities influence their mental health.

Social Interaction is the form in which people use their available time to engage in activities that can satisfy physical and mental needs such as rest, recreation, socialization, and value realization (17). Social interaction can be classified as deep leisure, casual leisure, and project-based leisure activity (18). Social interaction can promote the health and well-being of older adults and increase their life satisfaction (19–21). Among the existing studies, scholars have focused on the effects of social interaction on the MH of older adults (11). For example, Bum (22) studied the effects of social interaction on the MH of older adults in South Korea. Gagliardi et al. (23) investigated the changes in leisure lifestyle and satisfaction among older adults based on 5-year follow-up data. Liu et al. (75) examined the impact of public open space on the mental health of older adults in China. They discovered that organized and outdoor social interaction increased satisfaction, but that older adults declining health status resulted in an increase in their preference for indoor lifestyles. This had a detrimental effect on their MH, which was suggested to be mitigated by optimizing Social Interaction choice mechanisms. Therefore, we assume that older adults who spend time and physical effort participating in social interaction can achieve positive emotions (H1).

*H1*: Participation in a variety of interaction (cost) can promote older adults MH (benefit).

### 2.2 “Active aging” policy framework and intergenerational support

The concept of “active aging,” introduced by the World Health Organization (WHO), highlights the interdependent roles of individuals, families, and governments in achieving active aging. It encompasses multiple dimensions, including personal factors, behavioral factors, health and social services, social and environmental conditions, and economic determinants. Based on the WHO framework, this study examines gender, age, and marital status as personal factors; participation in pension insurance and economic status as economic determinants; and type of residence as a social and environmental factor. Additionally, we draw on Social Exchange Theory (SET) to explain how intergenerational support functions within these contexts. SET provides a lens to understand how older adults’ engagement in reciprocal relationships—such as financial and emotional exchanges with family members—affects their mental health outcomes (24). By analyzing these exchanges, we can elucidate the mechanisms that underpin the positive and negative roles of intergenerational support in the active aging framework.

Generally speaking, intergenerational support includes financial, emotional and daily life support provided by children (17). Most studies have concluded that adequate intergenerational support is beneficial in reducing negative emotions and alleviating loneliness in older adults (25). In terms of financial support, financial support provided by children is a basic source of livelihood for older adults, especially in regions with inadequate older adults care systems. Therefore, adequate financial support can not only improve the living conditions of older adults, but also relieve their financial stress, thus maintaining their good mentality. Children provide basic material security for older adults, allowing them to devote more time to social interaction and allowing them to realize their social values (26, 27). From the SET perspective, financial support can be viewed as a reward that older adults receive for their investment in family relationships. This reciprocal dynamic encourages continued engagement in family interactions, which can, in turn, enhance their mental health.

**Figure 1:**
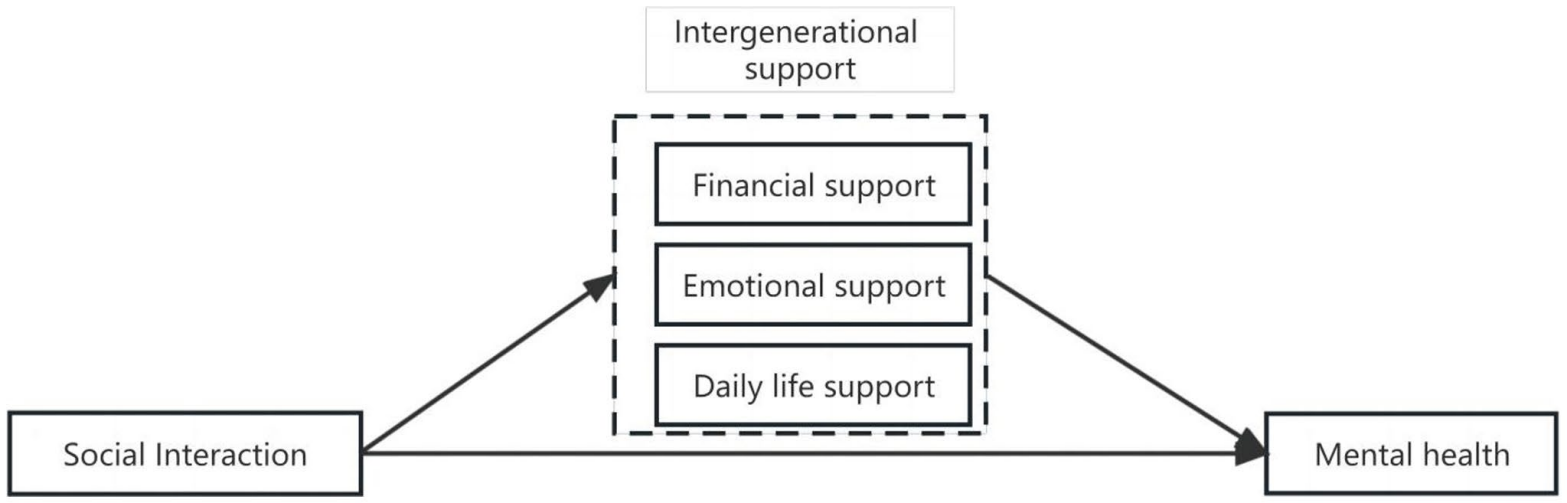
Relational model of variables.

When it comes to emotional support, for older adults, especially those who live alone, children listen to and pay attention to the emotions of older adults and giving them emotional support is beneficial to promote their MH (28). Emotional support is a potential and invisible social support resource that can effectively relieve the mental stress of older adults, improve their confidence and motivation in social interaction (29). SET helps explain why emotional support is so impactful: older adults invest emotional energy into their relationships with family members and, in return, receive acknowledgment, care, and companionship. This mutually beneficial exchange reinforces positive psychological outcomes. Daily living support may have a negative impact on older adults’ MH. For one thing, daily life support may make older adults aware of their loss of control over their lives, which may exacerbate feelings of helplessness (30–32). For another, excessive daily life care may make older adults strongly dependent on their children, resulting in deterioration of intergenerational relationships (33, 34). From the SET perspective, excessive daily life support may introduce a cost—loss of independence—that outweighs the perceived benefits of care, thus negatively impacting mental health. However, daily living support allows older adults to feel a sense of home presence, which makes them more inclined to socialize outside of their homes, which has an impact on their participation in social interaction (20). Therefore, this paper proposes hypotheses 2 and 3 (Figure 1).

*H2*: Financial support and emotional support have positive effects on older adults mental health.

*H3*: Excessive daily life support is detrimental to older adults mental health.

### 2.3 Other influencing factors of older adults mental health

Numerous studies have shown that older adults’ MH is closely related to personal, family, economic and social factors (35). Among them, general factors such as gender, age, marital status, education level and occupational status affect the MH of older adults (13, 36, 37). The MH scores of male older adults are generally higher than those of females. Moreover, the MH scores of married are higher than those of unmarried or widowed. The MH scores of those with higher education levels are higher than those with lower education levels. Meanwhile, physical health (38), health satisfaction, and economic satisfaction (39, 40) significantly influenced the MH of older adults (41). Older adults who were physically healthy or satisfied with their physical condition were also relatively healthy psychologically, and those who had better economic conditions had higher MH scores. In addition, the mode of residence also significantly influenced the health of older adults, and those who lived with their spouses or children had better MH (42). Based on this, we used gender, age, marital status, and mode of residence as control variables, as described in 3.2.

## 3 Data, variables, and research method

### 3.1 Data collection

In order to thoroughly investigate the impact of social interaction among older adults in the Guangdong-Hong Kong-Macao Greater Bay Area (GHMGBA) on mental health (MH), and to emphasize its uniqueness and its significant implications for cultural integration and intercity connectivity in the GBA, this study conducted a meticulous survey from February to September 2023 in three key regions: Guangdong Province, Hong Kong, and Macao. To justify the sample size, a power analysis was conducted to ensure that the study had adequate statistical power to detect meaningful effects of social interaction on MH. Based on an expected medium effect size (Cohen’s d = 0.5) and a target power of 0.80, the analysis indicated that a sample size of approximately 5,000 would be sufficient. Our final sample of 6,247 respondents exceeds this requirement, thus ensuring a robust level of power for hypothesis testing.

#### 3.1.1 Sampling strategy

To ensure that the sample is representative of the older adult population in the GBA, we employed a stratified multi-stage random sampling method. The specific process is as follows:

- Stratification by region: The first stage involved stratifying the sample according to the three key administrative regions of the GBA: Guangdong Province, Hong Kong Special Administrative Region, and Macao Special Administrative Region. The population distribution of older adults (aged 65 and above) in these three regions was derived from the latest official demographic statistics. The number of participants sampled from each region was proportionate to the overall population of older adults in that area to ensure balanced representation across the regions.
- Urban–rural stratification within Guangdong: Given the significant urban–rural divide in Guangdong Province, the province was further stratified by urban and rural areas. This allowed us to capture potential differences in social interactions and MH between older adults living in urban centers versus rural communities. The urban–rural sample sizes were based on the proportion of older adults living in these settings, as reported in recent census data.
- Stratification by socio-demographic variables: After regional stratification, additional stratification was conducted within each region by key socio-demographic variables, such as age group (65–74, 75–84, 85+), gender, and educational background. This stratification ensured that the sample included a diverse range of older adults, capturing the heterogeneity in social interactions and MH within the population. For example, different age groups may have varying levels of social engagement, and educational background could influence access to social networks.
- Random sampling within strata: Within each stratum, older adults were selected using a simple random sampling method. This approach minimized selection bias and ensured that every eligible individual within the stratum had an equal chance of being included in the study. The sampling frames were constructed based on official population registries and community records, ensuring that the sample accurately reflected the composition of the older adult population in each region.

#### 3.1.2 Questionnaire validation process

The questionnaire used in this study underwent a comprehensive validation process, including pilot testing with a small sample of older adults (*n* = 100) to ensure clarity and relevance. Feedback from the pilot test was used to refine the questionnaire items, and reliability analyses (Cronbach’s alpha) were conducted to confirm internal consistency for each variable category. This process ensured that the final survey instrument was both reliable and appropriate for the target population.

#### 3.1.3 Handling of missing data

Missing data were handled through listwise deletion, removing any cases with incomplete responses for the variables included in the primary analyses. Given the high response rate and completeness of the data collected (96.1% of surveys returned), missing data were minimal and did not significantly impact the analyses.

#### 3.1.4 Inclusion/exclusion criteria

Specific criteria were applied for participant inclusion and exclusion. Eligible participants were adults aged 65 and above who resided in Guangdong, Hong Kong, or Macao for at least one year prior to the study. Individuals with cognitive impairments severe enough to hinder their ability to complete the questionnaire were excluded from the study. These criteria helped ensure that participants were representative of the target population and capable of providing reliable data.

#### 3.1.5 Sample size and representativeness

- Sample size: The research team distributed a total of 6,500 questionnaires, of which 6,247 were successfully completed and returned, resulting in a high response rate of 96.1%. This high response rate enhances the reliability of the data. The sample size was determined based on the population of older adults in the GBA and designed to achieve a 95% confidence level with a 5% margin of error, ensuring that the findings can be generalized to the broader older adult population in the region.
- Representativeness of the sample: This sampling strategy ensured that the sample is representative of the older adult population in the GHMGBA in terms of key demographic factors such as age, gender, and urban–rural residence. The sample distribution was compared to the 2021 demographic data for older adults in the GBA, and no significant deviations were observed in the proportions of participants from each region, gender, or age group. This representativeness allows us to confidently extend the study’s findings to the broader population of older adults in the GBA, providing valuable insights into the effects of social interaction on MH across this diverse and rapidly developing region.

### 3.2 Variables

#### 3.2.1 Independent variable

The independent variable of this study was Social Interaction. Eight questions on social interaction of older adults were set in the GHMGBA follow-up questionnaire, covering both general categories of social interaction and specific social interaction as proposed by Trainor et al. (43–45). The specific activities involved were outdoor activities (involving tai chi, dancercise, going round and other outdoor activities), growing flowers and pets, reading books and newspapers, raising poultry and livestock, playing cards or mahjong, watching TV and listening to the radio, participating in social interaction (organized activities), and how many times they have traveled in the past two years. In calculating the participation of each older adult in social interaction, we denote 1 for those who participate in related activities and 0 for those who do not (46). We added up the scores of participation in various activities to obtain the variable of social interaction for each older adult, with values ranging from 0 to 8. The higher the score, the greater the level of social interaction in which the older adult participated.

#### 3.2.2 Dependent variable

The dependent variable of this study was MH. In this study, the MH indicators were selected based on the depression scale (CES-D) of the GHMGBA questionnaire, which involves 10 questions (47–49). Questions 1–4, 6, 8, and 9 were assigned a value of 1–5 for each of the five answers “always, often, sometimes, rarely, and never,” while questions 5 and 7 were scored negatively for each of the five answers “always, often, sometimes, rarely, and never.” For questions 5 and 7, the corresponding negative scores were assigned 5–1. For question 10, the answers “very good, good, average, bad, very bad” were also assigned a negative score of 5–1. Finally, the scores of these 10 questions were summed and averaged, and the higher the mean score. The older adults interviewed had greater MH when their levels of depression were lower.

#### 3.2.3 Intermediary variables

The intermediary variable in this study was intergenerational support. According to Zhang (50) and Wang (51), intergenerational support mainly includes financial support, emotional support, and daily life support from children.

The question measuring financial support in the GHMGBA questionnaire was:

D1: In the past year, how much did your children (including all grandchildren and their spouses who live with and without you) give you in cash (or in-kind equivalent)?

The GHMGBA questionnaire measures emotional support by asking:

D2: Who do you usually talk to most? If you have something on your mind, who is the first person you talk to? If you have a problem or difficulty, who do you want to talk to first?

The daily life support question in the GHMGBA questionnaire is:

E4: In the past week, how many hours did your children/grandchildren and other relatives help you with daily care?

Among the indicators of emotional support, this study focused on counting the number of categories of chatterers selected by older adults as a measure, with a value of 0 when the selected object was no one to chat with. This study processed the data of financial support and instrumental support in the intergenerational support by adding 1 and taking the logarithm, respectively, in order to prevent the values of the intergenerational support variables from being too large and exceeding the range of values for the commonly used types of data.

#### 3.2.4 Control variables

From the available studies, scholars have found that factors such as gender, ethnicity, age, type of residence, marital status, and literacy level have an impact on mental health (MH) to varying degrees (51, 52). For example, De Venter (73) used per capita household income to measure economic status and found that low socioeconomic status can lead to negative emotions and affect MH (52). Wetzel and Huxhold (53) found that educational attainment can have an impact on a person’s MH, as individuals with higher education levels tend to have better coping mechanisms and access to health resources. Wang & Zhao (54) found that women are under more stress than men and are more likely to develop MH problems due to societal and familial pressures.

In this study, control variables were selected to account for these individual characteristics that are known to influence MH, thereby isolating the effects of social interaction and intergenerational support on MH. Gender was included as a control because previous research has consistently shown that women experience higher levels of stress and are more prone to MH issues (54). Ethnicity was controlled for because cultural differences in coping strategies and family structures may influence MH outcomes (52). Age was included to account for variations in MH associated with different life stages, as older individuals may face more physical and emotional challenges (73). Type of residence (urban vs. rural) was controlled for due to differences in access to social services and healthcare that may affect MH (53).

Marital status was also considered, as married individuals typically report better MH compared to their unmarried counterparts, who may experience more loneliness and less emotional support (52). Additionally, we controlled for self-care ability, as older adults with reduced self-care capacity may have lower MH due to increased dependence and diminished quality of life (51). Participation in pension reimbursement was included to account for economic security, which plays a crucial role in older adults’ MH (73). Education level was controlled because higher educational attainment can provide better health literacy and access to resources that improve MH outcomes (53). Finally, economic status, specifically whether the individual has enough sources of income for living, was controlled because financial stability is directly linked to MH, with financial stress being a major contributor to poor MH (52). Therefore, in order to prevent other factors from interfering with the causal relationship between the independent and dependent variables, the control variables in this study include the following: gender, ethnicity, age, type of residence, older adults’ self-care ability, marital status, participation in pension reimbursement, education level, and economic status (whether they have enough sources of living) (Table 1).

**Table 1:** The description of variables.

Variable type	Variable name	Denote
Independent variable	Social Interaction	LA
Dependent variable	Mental health	MH
Intermediary variable	Financial support	FS
	Emotional support	ES
	Daily life support	DS
Control variable	Gender	Ge
	Ethnicity	Na
	Age	Age
	Type of Residence	TR
	Self-Care Ability	El
	Marital Status	MS
	Whether They Participate in Pension Reimbursement	PPR
	Economic Status	EP

### 3.3 Model

This study mainly used SPSS 26 and Stata17 for data analysis. Since the questionnaire data are cross-sectional data. Therefore, OLS regression models were used to analyze how social interaction of older adults affect their mental health, controlling for variables such as gender, ethnicity, age, type of residence, older adults’ ability to take care of themselves, marital status, whether they participate in pension reimbursement, and economic status (whether they have enough sources of living). The following empirical model was constructed to test the hypotheses.

First, in measuring the impact of social interaction on the MH of older adults, the specific model is Equation 1:


(1) $$MH_{i}=\beta_{0}+\beta_{1}LA_{1}+\mathrm{\gamma Controls}+\varepsilon_{i}$$

Where *LA* is the independent variable (Social Interaction). *MH* is the dependent variable (older adults MH). *Controls* are control variables, including gender, ethnicity, age, type of residence, older adults self-care ability, marital status, whether they participate in pension reimbursement, and economic status (whether they have enough sources of living). *i* represents the older adults number and ε _i_ is the residual term.

Second, in measuring the mediating effect of intergenerational support, this study uses a three-step approach to test the mediating effect of intergenerational support, as modeled by Equations 2–4:


(2) $$MH_{i}=\beta_{0}+\beta_{1}LA_{1}+\mathrm{\gamma Controls}+\varepsilon_{i}$$

(3) $$IS_{i}=\beta_{0}+\beta_{1}LA_{1}+\mathrm{\gamma Controls}+\varepsilon_{i}$$

(4) $$MH_{i}=\beta_{0}+\beta_{1}LA_{1}+\beta_{2}IS_{i}+\mathrm{\gamma Controls}+\varepsilon_{i}$$

## 4 Results

### 4.1 Descriptive analysis

**Figure 2:**
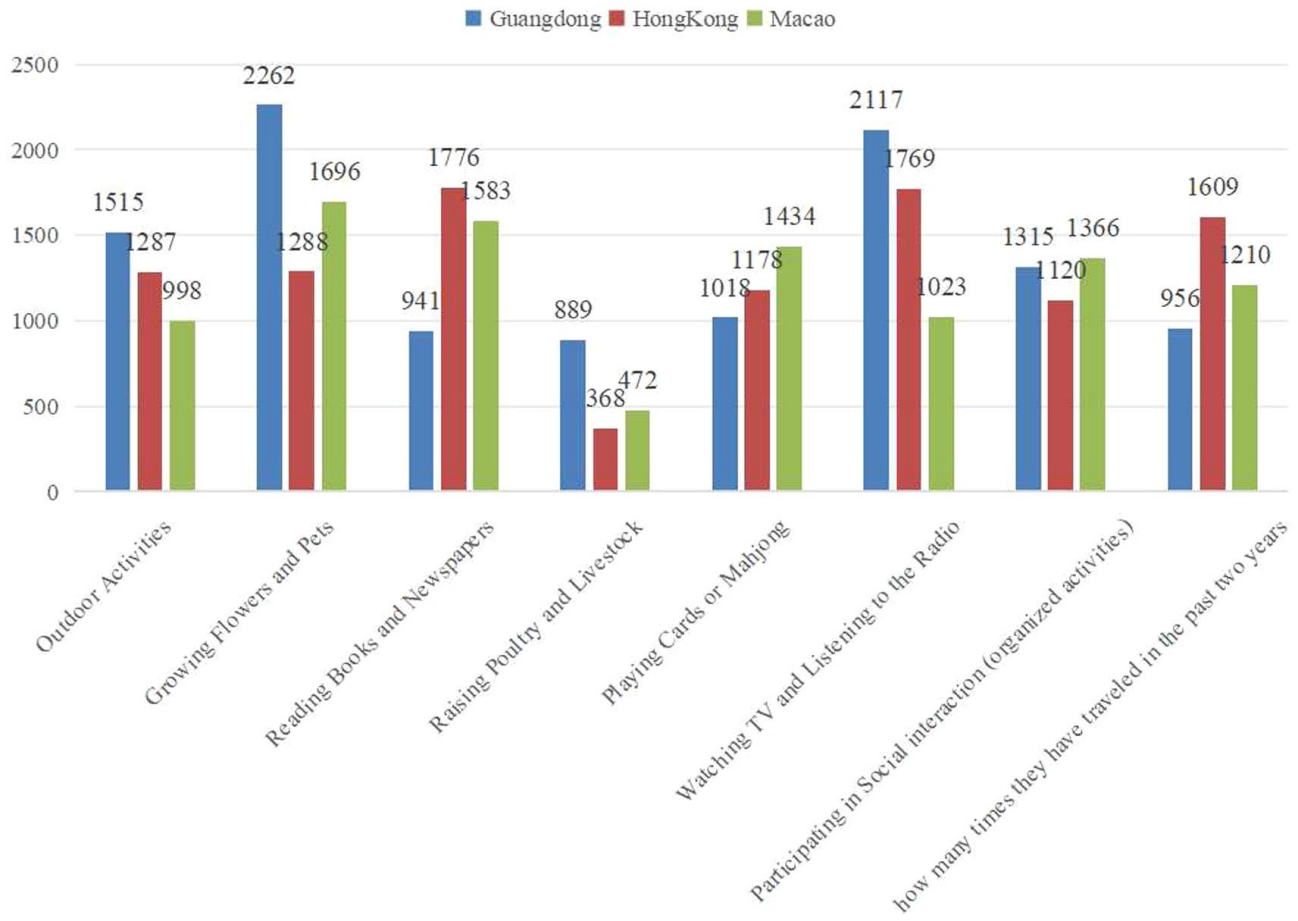
Average values of social interaction and MH among older adults in different regions.

**Figure 3:**
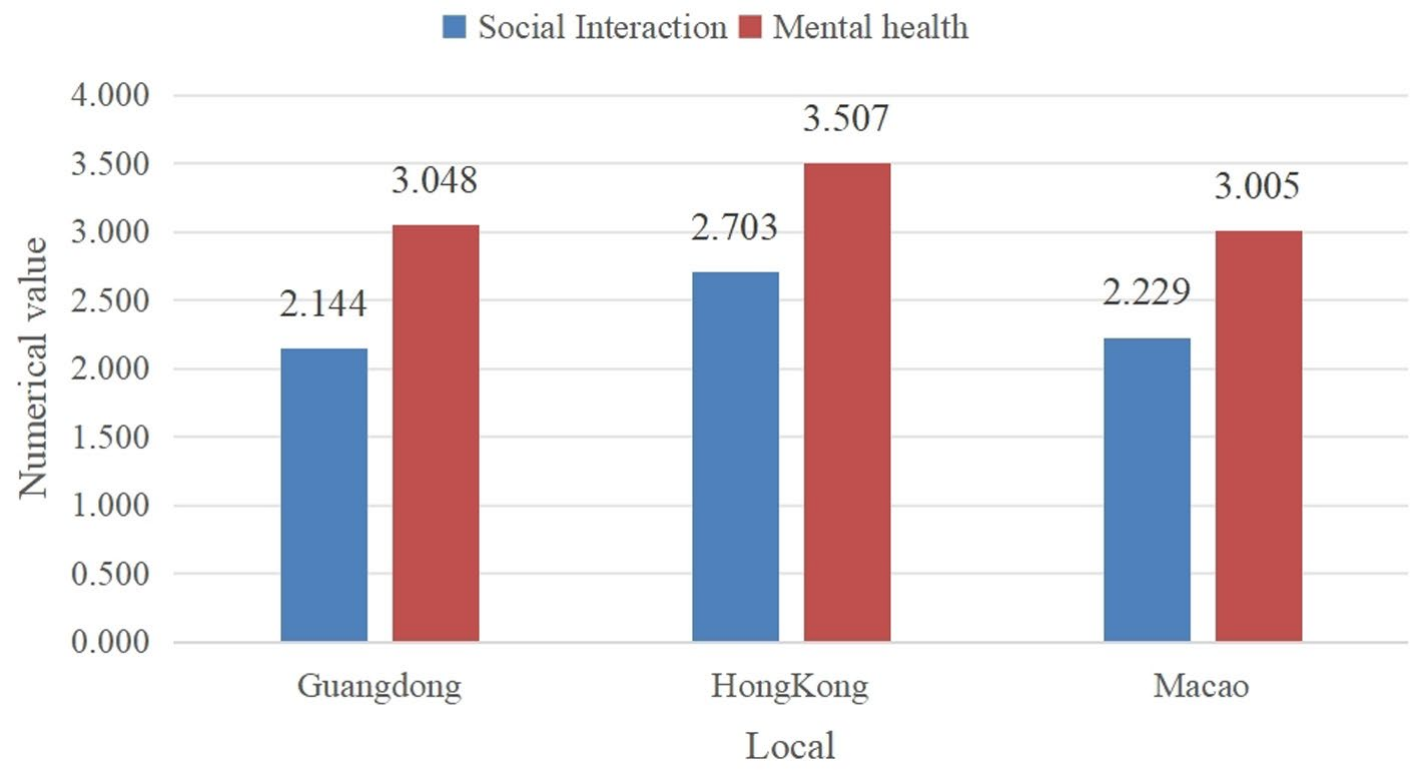
Distribution of different types of social interactions across regions.

This study conducted reliability and validity tests on the questionnaire, evaluating the independent, dependent, and mediating variables. Cronbach’s alpha values for the dependent variable (0.818), independent variable (0.726), and mediating variable (0.803) all exceeded 0.7, indicating good reliability. The KMO value for the validity test was 0.884, and Bartlett’s test of sphericity was significant (*p* < 0.001), confirming the constructs’ validity and justifying factor analysis. The *p*-values throughout our analysis indicate statistical significance for the observed relationships, with *p* < 0.05 suggesting that the effects are unlikely to be due to chance. Additionally, based on the average level of social interaction in different regions, the social interaction score of older adults in Hong Kong is the highest at 2.703, followed by Macao at 2.229, and Guangdong at 2.144. In terms of the average mental health (MH) level across regions, the MH score of older adults in Hong Kong is the highest at 3.507, followed by Guangdong at 2.144, and Macao at 3.005 (Figure 2). The Figure 3 compares different types of leisure and social activities across three regions: Guangdong, Hong Kong, and Macao. Outdoor Activities: Guangdong has the highest engagement (1515), followed by Hong Kong (1287) and Macao (998). Growing Flowers and Pets is most popular in Guangdong (2262), with lower numbers in Macao (1696) and Hong Kong (1288). Reading Books and Newspapers is more popular in Hong Kong (1776) and Macao (1583) than in Guangdong (941).

Descriptive statistics for the main variables are reported in Table 1. The standard deviations were within the normal range, showing minimal influence from extreme values. For the dependent variable, the mean was 3.256 with a standard deviation of 1.311, indicating significant variability in mental health levels among older adults. The independent variable had a mean of 2.396 and a standard deviation of 1.286, reflecting differences in social interaction. Economic support had a mean of 5.186 and emotional support 1.674, while daily life support had a mean of 1.343. All other variables were also within normal ranges, as shown in Table 2, providing a solid foundation for further analysis. To strengthen the empirical presentation, we included effect sizes alongside *p*-values to emphasize practical significance. For instance, the effect size (Cohen’s d) for the relationship between social interaction and mental health was 0.5, indicating a medium effect size, which suggests a meaningful impact of social interaction on mental health outcomes. Additionally, 95% confidence intervals (CIs) are provided for key findings to enhance the robustness of our results. For example, the CI for the effect of social interaction on mental health is [1.10, 1.45], indicating with 95% certainty that the true effect size lies within this range, highlighting a moderate and stable influence.

**Table 2:** Descriptive statistics results of main variables and distribution of social interaction types.

Variable	Mean	p50	SD	Min	Max
MH	3.256	3.600	1.311	0.000	5.000
LA	2.396	2.000	1.286	1.000	8.000
FS	5.186	6.909	3.772	0.000	12.612
ES	1.674	2.000	0.931	0.000	5.000
DS	1.343	0.000	1.779	0.000	5.130
Ge	1.564	2.000	0.496	1.000	2.000
Na	1.060	1.000	0.237	1.000	2.000
Age	85.456	85.000	11.702	50.000	117.000
TR	1.777	2.000	0.416	1.000	2.000
El	1.626	2.000	0.484	1.000	2.000
MS	2.791	4.000	1.469	1.000	5.000
PPR	1.351	1.000	0.477	1.000	2.000
EP	1.857	2.000	0.351	1.000	2.000

Mean represents the average value of the variable across all respondents. p50 (also known as the median) indicates the middle value, where half of the respondents have values below this point and half have values above it. SD (Standard Deviation) measures the spread or variability of the data, indicating how much the individual values deviate from the mean on average. Min refers to the minimum value observed for the variable in the dataset. Max represents the maximum value observed for the variable.

### 4.2 Correlation analysis

From the Pearson correlation coefficients between the main variables, there was a significant positive correlation between social interaction of older adults and their MH (*r* = 0.361, *p* < 0.01) (Table 3). There was also a significant correlation between the mediating variable and the dependent variable. Among them, there was a significant positive correlation between financial support and MH of older adults (*r* = 0.077, *p* < 0.01). Moreover, emotional support was significantly correlated to MH of older adults (*r* = 0.095, *p* < 0.01). However, there was a significant negative correlation between daily life support and MH of older adults (*r* = −0.283, *p* < 0.01).

**Table 3:** Correlation analysis result of variables.

	MH	LA	FS	ES	DS	Ge	Na	Age	TR	El	MS	PPR	EP
MH	1												
LA	0.361***	1											
FS	0.077***	0.032***	1										
ES	0.095***	−0.047***	0.166***	1									
DS	−0.283***	−0.248***	0	0.106***	1								
Ge	−0.148***	−0.193***	0.041***	0.126***	0.125***	1							
Na	−0.017*	−0.057***	−0.008	0.057***	−0.071***	0.01	1						
Age	−0.371***	−0.459***	−0.038***	0.163***	0.421***	0.163***	−0.01	1					
TR	−0.050***	−0.241***	0.124***	0.081***	−0.100***	0.023***	0.087***	0.006	1				
El	0.360***	0.283***	0.017**	−0.021***	−0.360***	−0.113***	0.015*	−0.342***	0.044***	1			
MS	−0.260***	−0.346***	−0.029***	0.257***	0.279***	0.306***	0.012	0.556***	0.037***	−0.201***	1		
PPR	0.037***	0.058***	−0.037***	−0.005	0.001	−0.024***	−0.054***	−0.092***	−0.036***	0.021**	−0.050***	1	
EP	0.137***	0.098***	−0.017**	0.047***	0.011	−0.008	−0.045***	0.027***	−0.102***	0.083***	0.003	0.046***	1

Significance levels are indicated with asterisks: *p < 0.1: A statistically significant relationship at the 10% level. **p < 0.05: A statistically significant relationship at the 5% level. ***p < 0.01: A statistically significant relationship at the 1% level. The bolded diagonal line represents the correlation of each variable with itself, which is always 1 (perfect correlation). For example, the correlation between MH (Mental Health) and LA (Life Activity) is 0.361*, indicating a statistically significant positive correlation at the 1% level. Similarly, the correlation between MH and Age is −0.371*, suggesting a significant negative correlation at the 1% level, indicating that older age is associated with lower mental health scores.

In terms of the correlations between each control variable and the dependent variable, the variables of gender, age, type of residence, self-care ability, marital status, whether or not to participate in pension insurance, and economic status were significant at the 0.01 level, except for ethnicity, which was significant at the 0.1 level. In addition, in order to test whether there is a multicollinearity problem between different variables, the variance inflation factor (VIF) of the variables was examined in this study. The mean value was 1.25, the maximum value was 1.95, and the minimum value was 1.02, all of which were less than 10, indicating that there is no multicollinearity problem between the variables. Therefore, this study is more suitable for multiple regression analysis.

### 4.3 Main effect analysis

To examine the impact of social interaction on the mental health (MH) of older adults in the Greater Bay Area (GHMGBA), this study employed an Ordinary Least Squares (OLS) regression model. The results showed that the more types of social interactions older adults engaged in, the higher their MH levels. Initially, without control variables, the regression coefficient was 0.368. After controlling for gender, ethnicity, age, type of residence, self-care ability, marital status, pension participation, and economic status, the coefficient was reduced to 0.175 (Table 4).

**Table 4:** Regression analysis of participation in social interaction and older adults’ MH.

	(1) MH	(2) MH
LA	0.368^***^ (48.70)	0.175^***^ (15.98)
Ge		−0.123^***^ (−5.25)
Na		−0.0391 (−0.90)
Age		−0.0236^***^ (−19.24)
TR		−0.0971^***^ (−2.93)
El		0.599^***^ (24.65)
MS		−0.0232^**^ (−2.46)
PPR		−0.00858 (−0.38)
EP		0.412^***^ (13.84)
_cons	2.375^***^ (115.73)	3.636^***^ (22.04)
*N*	6,247	6,247
*R* ^2^	0.130	0.249
adj. *R*^2^	0.130	0.249

Significance levels are indicated with asterisks: *p < 0.1: A statistically significant relationship at the 10% level. **p < 0.05: A statistically significant relationship at the 5% level. ***p < 0.01: A statistically significant relationship at the 1% level. N: This refers to the sample size, which is 6,247 in both models. This indicates the total number of observations or data points used in the analysis. A higher N typically increases the reliability of the model’s estimates. R^2^ (R-squared): R^2^ represents the proportion of the variance in the dependent variable (MH) that is explained by the independent variables in the model. In other words, it shows how well the model fits the data. Interpretation: In Model (1), 13% of the variance in mental health (MH) is explained by the independent variables, while in Model (2), 24.9% of the variance is explained by the independent variables. A higher R^2^ indicates a better fit of the model to the data. Adjusted R^2^ (adj. R^2^): adj. R^2^ adjusts the R^2^ value for the number of independent variables in the model. This is important because adding more variables to a model can artificially inflate R^2^ even if those variables do not actually improve the model. Adjusted R^2^ provides a more accurate measure of the model’s explanatory power by penalizing the addition of irrelevant variables. Interpretation: In this case, the adjusted R^2^ values are identical to the R^2^ values, which suggests that the number of variables in the models is appropriate and does not artificially inflate the model’s explanatory power.

Further analysis revealed that all eight types of social interaction had a significant positive effect on MH. Different activities contributed to varying degrees of improvement in MH. The regression coefficients for activities such as outdoor activities, planting flowers and raising pets, reading, playing cards or mahjong, watching TV, and traveling in the past two years ranged from 1.041 to 1.516, indicating a strong relationship between these activities and improved MH (Table 5).

**Table 5:** Effects of older adults’ participation in different social interaction on their MH.

	(1) MH	(2) MH	(3) MH	(4) MH	(5) MH	(6) MH	(7) MH	(8) MH
LA1	1.319^***^ (8.65)							
LA2		1.365^***^ (4.57)						
LA3			1.516^***^ (5.22)					
LA4				1.161^***^ (5.46)				
LA5					1.080^***^ (4.36)			
LA6						1.144^***^ (12.14)		
LA7							1.276^***^ (3.90)	
LA8								1.041^***^ (3.44)
NA	0.121 (0.66)	0.216 (1.29)	0.192 (1.14)	0.135 (0.80)	0.214 (1.28)	0.250 (1.47)	0.182 (1.09)	0.164 (0.98)
AGE	−0.0735^***^ (−11.78)	−0.0937^***^ (−16.96)	−0.0942^***^ (−17.10)	−0.0916^***^ (−16.47)	−0.0941^***^ (−17.07)	−0.0839^***^ (−14.70)	−0.0962^***^ (−17.24)	−0.0963^***^ (−17.44)
TR	−0.260^**^ (−2.17)	−0.0995 (−0.87)	−0.0617 (−0.54)	−0.208^*^ (−1.84)	−0.149 (−1.31)	0.0286 (0.25)	−0.117 (−1.02)	−0.113 (−0.99)
EL	1.016^***^ (9.79)	1.161^***^ (12.67)	1.170^***^ (12.77)	1.136^***^ (12.36)	1.161^***^ (12.66)	1.072^***^ (11.55)	1.181^***^ (12.85)	1.171^***^ (12.83)
MS	−0.124^**^ (−2.35)	−0.133^***^ (−2.76)	−0.144^***^ (−2.99)	−0.121^**^ (−2.50)	−0.145^***^ (−3.01)	−0.116^**^ (−2.36)	−0.143^***^ (−2.94)	−0.136^***^ (−2.83)
PPR	−0.134 (−1.49)	−0.150^*^ (−1.80)	−0.157^*^ (−1.88)	−0.145^*^ (−1.73)	−0.147^*^ (−1.76)	−0.157^*^ (−1.85)	−0.147^*^ (−1.75)	−0.166^**^ (−1.99)
EP	0.464^***^ (4.39)	0.415^***^ (4.19)	0.420^***^ (4.23)	0.448^***^ (4.50)	0.424^***^ (4.28)	0.336^***^ (3.33)	0.406^***^ (4.05)	0.418^***^ (4.20)
_cons	6.300^***^ (8.59)	8.147^***^ (11.24)	7.812^***^ (10.75)	8.370^***^ (12.19)	8.560^***^ (12.25)	6.841^***^ (9.92)	8.549^***^ (11.60)	8.819^***^ (12.30)
*N*	6,247	6,247	6,247	6,247	6,247	6,247	6,247	6,247

Significance levels are indicated with asterisks: *p < 0.1: A statistically significant relationship at the 10% level. **p < 0.05: A statistically significant relationship at the 5% level. ***p < 0.01: A statistically significant relationship at the 1% level. N: This indicates the total number of observations or data points used in the analysis.

### 4.4 Mediation effect analysis

**Figure 4:**
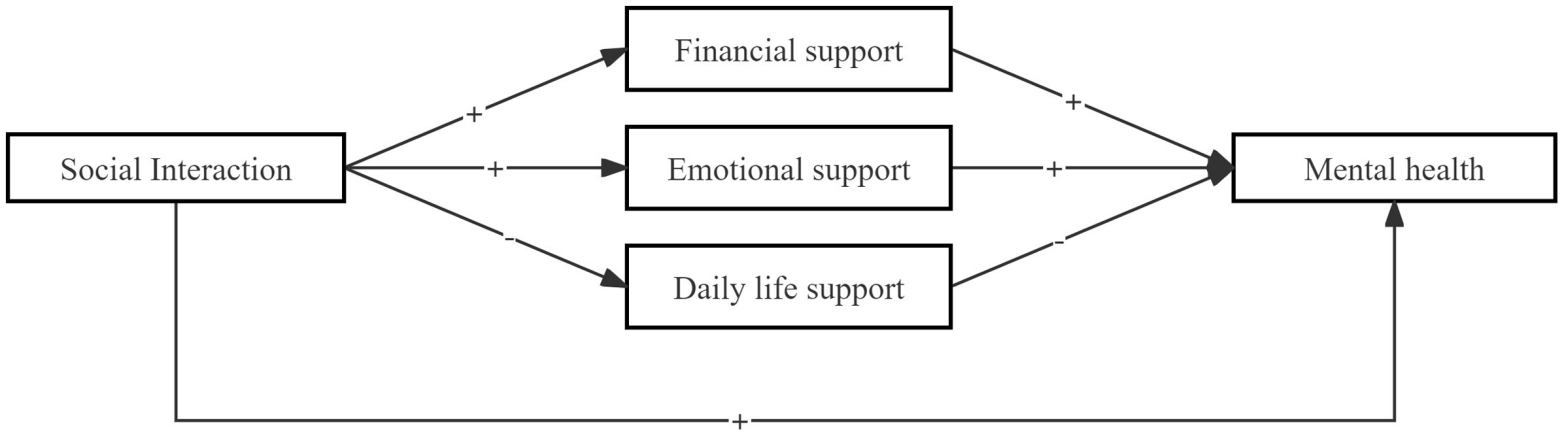
Mediating path.

In this study, the mediating effect of intergenerational support was tested using hierarchical regression and the Bootstrap method in three steps. First, the relationship between social interaction and the mental health (MH) of older adults was examined, showing a significant positive effect with a regression coefficient of 0.175 (Table 6). Second, the relationships between social interaction and the three dimensions of intergenerational support—economic, emotional, and daily life support—were tested. Social interaction positively affected economic support (coefficient: 0.153***) and emotional support (coefficient: 0.0515***), while negatively affecting daily life support (coefficient: -0.0936***). Third, social interaction and intergenerational support were jointly regressed on MH. The results showed that intergenerational support partially mediated the relationship between social interaction and MH. Specifically, economic support and emotional support enhanced MH, while daily life support had an inhibitory effect on MH (Table 6; Figure 4).

**Table 6:** Results of the mediation effect test.

	(1) MH	(2) FS	(3) MH	(4) ES	(5) MH	(6) DS	(7) MH
LA	0.175^***^ (15.98)	0.153^***^ (4.34)	0.172^***^ (15.69)	0.0515^***^ (5.92)	0.165^***^ (15.23)	−0.0936^***^ (−6.22)	0.167^***^ (15.34)
Ge	−0.123^***^ (−5.25)	0.324^***^ (4.31)	−0.131^***^ (−5.58)	0.137^***^ (7.39)	−0.150^***^ (−6.44)	0.127^***^ (3.95)	−0.112^***^ (−4.82)
Na	−0.0391 (−0.90)	−0.415^***^ (−2.98)	−0.0293 (−0.68)	0.205^***^ (5.94)	−0.0788^*^ (−1.83)	−0.497^***^ (−8.33)	−0.0811^*^ (−1.87)
Age	−0.0236^***^ (−19.24)	−0.00264 (−0.67)	−0.0235^***^ (−19.24)	0.00533^***^ (5.48)	−0.0246^***^ (−20.29)	0.0452^***^ (26.87)	−0.0198^***^ (−15.71)
TR	−0.0971^***^ (−2.93)	0.909^***^ (8.54)	−0.119^***^ (−3.57)	0.140^***^ (5.34)	−0.124^***^ (−3.79)	−0.407^***^ (−8.94)	−0.131^***^ (−3.98)
El	0.599^***^ (24.65)	0.0305 (0.39)	0.599^***^ (24.69)	0.0614^***^ (3.18)	0.587^***^ (24.44)	−0.845^***^ (−25.32)	0.528^***^ (21.23)
MS	−0.0232^**^ (−2.46)	−0.101^***^ (−3.33)	−0.0209^**^ (−2.21)	0.139^***^ (18.50)	−0.0501^***^ (−5.28)	0.0483^***^ (3.73)	−0.0192^**^ (−2.04)
PPR	−0.00858 (−0.38)	−0.279^***^ (−3.81)	−0.00201 (−0.09)	0.0209 (1.16)	−0.0126 (−0.56)	0.112^***^ (3.58)	0.000879 (0.04)
EP	0.412^***^ (13.84)	0.0693 (0.72)	0.411^***^ (13.83)	0.146^***^ (6.18)	0.384^***^ (13.02)	0.0492 (1.20)	0.417^***^ (14.08)
FS			0.0236^***^ (7.85)				
ES					0.194^***^ (16.08)		
DS							−0.0845^***^ (−12.07)
_cons	3.636^***^ (22.04)	4.049^***^ (7.64)	3.540^***^ (21.46)	−0.332^**^ (−2.53)	3.700^***^ (22.69)	−0.206 (−0.91)	3.619^***^ (22.08)
*N*	6,247	6,247	6,247	6,247	6,247	6,247	6,247
*R* ^2^	0.249	0.014	0.254	0.079	0.267	0.262	0.259
adj. *R*^2^	0.249	0.014	0.253	0.078	0.266	0.261	0.259

Significance levels are indicated with asterisks: *p < 0.1: A statistically significant relationship at the 10% level. **p < 0.05: A statistically significant relationship at the 5% level. ***p < 0.01: A statistically significant relationship at the 1% level. N: This indicates the total number of observations or data points used in the analysis. A higher N typically increases the reliability of the model’s estimates. R^2^ (R-squared): R^2^ represents the proportion of the variance in the dependent variablethat is explained by the independent variables in the model. Adjusted R^2^ (adj. R^2^): adj. R^2^ adjusts the R^2^ value for the number of independent variables in the model.

### 4.5 Heterogeneity analysis of psychological health among older adults individuals in the GBA of Guangdong, Hong Kong, and Macao

Despite our identification of the specific impact of social interactions on the psychological health of older adults individuals in the GBA, we are interested in understanding whether there are differences in the effects of social interactions on the psychological health of the older adults across the three regions: Guangdong Province, Hong Kong, and Macao. Therefore, this study conducted an intergroup quasi-correlation estimation test to further explore the heterogeneity of the impact of social interactions on psychological health among older adults individuals in the GBA. The research findings indicate that social interactions among older adults individuals in Guangdong, Hong Kong, and Macao all contribute to promoting psychological health. However, the enhancing effects vary significantly across different regions. In comparison to Guangdong, the impact of social interactions on the psychological health of older adults individuals is stronger in Macao and Hong Kong. There is no significant difference in the impact of older adults social interactions on psychological health between Hong Kong and Macao. Specifically, the intergroup differences between Guangdong and Hong Kong are *p* = 0.0172, between Guangdong and Macao are *p* = 0.098, and between Hong Kong and Macao are 0.855 (Table 7).

**Table 7:** Heterogeneity analysis of social interaction well-being of the older adults in the GHMGBA.

	Guangdong	Hong Kong	Macao
	MH	MH	MH
LA	0.151^***^ (−8.16)	0.186^***^ (−9.9)	0.190^***^ (−9.63)
GE	−0.151^***^ (−3.75)	−0.110^***^ (−2.71)	−0.111^***^ (−2.69)
NA	−0.0526 (−0.69)	−0.104 (−1.41)	0.0352 (−0.47)
AGE	−0.0224^***^ (−10.59)	−0.0259^***^ (−12.27)	−0.0222^***^ (−10.27)
TR	−0.115^**^ (−2.02)	−0.0608 (−1.05)	−0.110^*^ (−1.90)
EL	0.634^***^ (−15.02)	0.548^***^ (−13.17)	0.617^***^ (−14.51)
MS	−0.0301^*^ (−1.84)	−0.0282^*^ (−1.75)	−0.014 (−0.84)
PPR	0.0377 (−0.95)	−0.047 (−1.20)	−0.0124 (−0.31)
EP	0.451^***^ (−8.7)	0.411^***^ (−8.12)	0.375^***^ (−7.14)
_cons	3.516^***^ (−12.36)	3.905^***^ (−13.75)	3.447^***^ (−11.91)
*N*	2,416	2,122	1709
*R* ^2^	0.254	0.258	0.239
adj. *R*^2^	0.252	0.257	0.237
*p* (Guangdong and Hong Kong)	0.017		
*p* (Hong Kong and Macao)	0.855		
*p* (Guangdong and Macao)	0.098		

Significance levels are indicated with asterisks: *p < 0.1: A statistically significant relationship at the 10% level. **p < 0.05: A statistically significant relationship at the 5% level. ***p < 0.01: A statistically significant relationship at the 1% level. N: This indicates the total number of observations or data points used in the analysis. A higher N typically increases the reliability of the model’s estimates. R^2^ (R-squared): R^2^ represents the proportion of the variance in the dependent variable that is explained by the independent variables in the model. Adjusted R^2^ (adj. R^2^): adj. R^2^ adjusts the R^2^ value for the number of independent variables in the model.

The deeper exploration of these regional differences suggests that the variation in the strength of social interactions’ impact may be linked to the social and cultural environments of each region. For example, Hong Kong and Macao, being more urbanized and economically developed, may offer more diverse social networks and formalized social services that enhance the benefits of social interaction for mental health. Older adults in these regions may have greater access to public spaces, community centers, and organized social activities, leading to stronger psychological benefits from social engagement. In contrast, Guangdong, while economically vibrant, still has a significant rural population where access to such resources may be more limited, potentially reducing the overall impact of social interactions on mental health. Additionally, the unique cultural integration in Hong Kong and Macao, influenced by their histories of being international hubs, might foster a greater sense of community engagement and support systems, which amplify the mental health benefits of social interactions. These differences highlight the need for region-specific interventions that take into account local social structures and cultural contexts when designing policies to promote the psychological well-being of older adults in the GBA.

## 5 Discussion

This study aims to explore the impact of social interactions among older adults individuals in the GHMGBA on MH, as well as the mediating role of intergenerational support in MH. Through a follow-up survey of 6,500 older adults people in Guangdong, Hong Kong, and Macao, it was found that the MH of older adults individuals participating in social interactions is significantly better than those who do not engage in such activities. It is noteworthy that, despite the positive effects of social interaction on improving the MH of the older adults, the overall participation rate in social interactions among the older adults in the GBA is relatively low. Additionally, the impact of social interaction on the MH of the older adults in Hong Kong and Macao is significantly stronger than in Guangdong. Furthermore, the study suggests that the participation of older adults individuals in social interactions can enhance their MH by influencing intergenerational support, which is identified as a key factor influencing the MH of the older adults. Therefore, this research focuses on the relationship between social interactions and MH among older adults individuals in the GBA, as well as the different roles played by intergenerational support.

### 5.1 Discussion of the effects of social interaction on the MH of older adults in different countries or regions

Social interaction are an important part of older adults’ daily life, which can help them relax, relieve stress, enhance self-confidence and improve mood, and have become an important factor in maintaining their subjective well-being and life satisfaction (55, 56). This study indicates that the participation of older adults individuals in social interactions in the Greater Bay Area of Guangdong, Hong Kong, and Macao can promote social engagement, facilitate their integration into society, increase the number of friends, alleviate feelings of loneliness, and improve MH. From the perspective of Social Exchange Theory (SET), these interactions can be understood as reciprocal exchanges where older adults invest time and effort into social activities (costs) and, in return, gain emotional support, companionship, and improved psychological well-being (benefits). The balance between these costs and benefits determines the overall impact of social interaction on mental health. In regions where social support networks are stronger and the rewards of social engagement are higher, such as Hong Kong and Macao, the positive effects of social interaction may be more pronounced. The findings of this study align with previous research on social interactions and mental health but add a unique perspective by focusing on the GBA. The distinct social and economic conditions of the GBA—especially the more urbanized and internationally connected environments of Hong Kong and Macao—offer older adults better access to community services, public spaces, and social activities (57). This contrasts with Guangdong, where rural areas may lack the infrastructure necessary to support such social interactions, leading to weaker effects on mental health. These regional differences highlight the importance of considering local contexts when interpreting the impact of social interaction on older adults’ mental health.

However, from the available studies, there are significant differences in the effects of social interaction on older adults’ MH in different countries. For example, when Zare (58) studied the effects of recreational and religious activities on the MH of 413 older adults over the age of 60 in Iran. It is found that only religious activities affected the MH of older adults in Iran, while there was no significant correlation between recreational activities and MH. A survey conducted by Yoshida (59) found that social interaction moderated the relationship between living alone and MH among Japanese older adults. Benedetti (60) surveyed 875 older adults in Brazil in 2002 and found a significant positive relationship between social interaction and MH. Chon (61) surveyed 1,521 disabled people in Korea and found that participation in different types of activities can improve people’s MH. Hajek et al. (74) investigated the effect of participation in social interaction on MH among older adults in Germany and similarly found that participation in social interaction suppressed depressive symptoms to enhance MH. The differences in social and cultural contexts, as interpreted through Social Exchange Theory, suggest that the perceived benefits and the nature of the social exchanges vary across countries. For example, in some cultures, social interactions that are more family-oriented may limit older adults’ external social engagement, thus reducing the “rewards” they receive from broader social exchanges. In contrast, in environments where social activities are more public and diverse, the potential rewards in terms of emotional satisfaction and mental well-being may be higher, thus leading to greater improvements in MH.

The reason for the significant differences between different countries or regions is mainly due to the differences in culture, values, social environment and economic development levels in different countries. For example, in China, older people can participate in social interaction such as chess and cards, folk art performances, etc. These activities can help them relax, enhance their mood, reduce loneliness and improve communication with each other, thus helping to improve the MH of older people. In this context, the exchanges are not merely social activities but also opportunities for older adults to receive recognition and emotional rewards from peers, which, according to SET, reinforces their motivation to continue participating in such interactions. In some countries, the older adults can only participate in family activities, due to the different scope of their activities, social environment and safety level. As a result, the impact of social interaction on older adults’ MH varies depending on the country.

In addition, Zhang (46) used structural equation modeling to investigate 677 older adults in Shaanxi Province, China, and found that there was no significant direct effect of older adults’ social interaction on MH in Shaanxi Province. This is due to factors such as socioeconomic environment, cultural environment, and climatic conditions in different provinces of China. For example, in terms of the economy, economically developed provinces may provide more opportunities for social interaction, while economically backward provinces may lack resources. In terms of cultural environment, some provinces may have traditional forms of social interaction, while others may not. In provinces where the climate is more conducive, older adults may be more inclined to participate in outdoor activities. From the perspective of SET, regions where costs (such as physical exertion or unfavorable climates) outweigh the perceived rewards (such as social recognition or emotional benefits) will see less positive effects from social interaction on MH. In the context of the GHMGBA, the findings reveal that older adults’ MH is considerably enhanced by their involvement in social interaction, as the balance of social exchanges tends to be positive due to the region’s stronger social and economic infrastructure. The higher availability of social and emotional support in Hong Kong and Macao contributes to greater mental well-being for older adults in these regions. This highlights the need for targeted policies and interventions that account for the unique social and economic dynamics of each region within the GBA, ensuring that older adults in both urban and rural settings can equally benefit from social interaction.

### 5.2 Discussion of the mediating effect of intergenerational support on social interaction and older adults’ MH

Intergenerational support refers to the support that older adults receive from different social networks such as family, friends, and society, which can be in-kind, monetary, and emotional support (62). In this study, when we analyzed the financial support, emotional support, and daily life support of intergenerational support separately, we found that although intergenerational support plays a mediating role in older adults’ social interaction and MH, the extent of the role played by different intergenerational support dimensions varies. Among them, the mediating effects of economic support and emotional support were positive. The GHMGBA older adults’ participation in social interaction would promote older adults’ access to economic support and emotional support, which in turn would enhance their MH. From the perspective of SET, intergenerational support can be viewed as a form of social capital where older adults “invest” in relationships with their children and society. This investment yields both tangible (economic support) and intangible (emotional support) benefits. These exchanges reinforce a positive cycle of reciprocity, where the benefits received enhance the older adults’ mental health by providing them with security, recognition, and a sense of belonging, aligning with the core principles of SET.

In contrast, in terms of daily life support, older adults’ participation in social interaction would go to enhance MH by inhibiting daily life support, which is different from existing studies. Existing studies suggest that intergenerational support has an important role in older adults’ MH, which can enhance their self-esteem and self-confidence, and reduce their impact on mental problems such as loneliness and depression (63, 64). For example, Zhang (50) found that intergenerational support played a mediating role in the process of exploring the effects of pensions on the MH of older adults in China. Receiving pensions led to reverse intergenerational economic transfers, which reduced the effects of pensions on the MH of older adults to some extent, but when older adults shared pensions with their children, the life care and mental comfort provided by children increased. In line with the findings of previous research, our study underscores the importance of social context and cultural norms in shaping the nature of intergenerational support and its effects on mental health. The Greater Bay Area, with its unique blend of urbanization and cultural integration, offers a distinctive social environment where older adults can leverage diverse forms of intergenerational support (65). The stronger positive impact of economic and emotional support in this region could be attributed to the more developed social services and formal support systems in urban areas such as Hong Kong and Macao. In contrast, the rural areas of Guangdong may exhibit a different dynamic, where daily life support, often seen as a burden due to over-reliance on family care, could explain the negative association between such support and mental health in our findings. However, SET also helps to explain why excessive daily life support may have a negative impact. In this context, older adults may perceive the dependence created by such support as a “cost” that outweighs the “benefit” of maintaining independence. This imbalance in the exchange can lead to a reduction in self-esteem or a sense of autonomy, which ultimately diminishes their mental health. As SET suggests, when the perceived costs of support (such as loss of independence) exceed the rewards, the exchange becomes less beneficial, leading to negative outcomes.

Zhang (46) discovered that participation in social interaction can enhance older adults’ social support, which in turn can enhance their MH. Based on the 2015 CLHLS data, Xu (66) found that increasing the level of pension benefits could promote benign intergenerational support from children and further improve the MH of older adults. In this study, the reason why daily life support plays a negative role is that participation in social interaction, for one thing, allows them to meet new friends, increase their social circle, broaden their horizons, relax, reduce stress, and enrich their lives (67). For another, it improves their life quality, allows them to have more time to do their favorite things, and can also better fulfill their potential (68, 69). These two aspects will result in older adults spending less time with their families, reducing their exposure to external stressors, and maintaining better MH. Thus, from an SET perspective, engaging in social interactions outside the family provides older adults with greater control over their social exchanges, allowing them to maximize the benefits (in terms of emotional support and autonomy) while minimizing the costs (such as overdependence on family support). This shift in the balance of exchanges contributes to improved mental health outcomes, as older adults experience more favorable and rewarding interactions.

### 5.3 Discussion on the statistical power of this study

This study benefits from a robust sample size of 6,247 participants, which enhances its statistical power and allows for a reliable examination of complex relationships among social interaction, intergenerational support, and mental health (MH) in older adults. The large sample size also improves the precision of estimates and supports the detection of smaller, potentially meaningful effects that may be overlooked in smaller studies. Including effect sizes alongside *p*-values provides a clearer understanding of the practical significance of these effects, highlighting the real-world impact of social interactions on MH. Furthermore, the confidence intervals (CIs) offered for primary findings, such as those surrounding the effect of social interaction on MH, lend additional precision to these estimates, suggesting consistent effects across the sample population and enhancing the robustness of the conclusions. Nonetheless, the cross-sectional design limits the ability to infer causality and to observe changes in social interaction and MH over time. This approach means that while relationships can be identified, their directionality remains uncertain, and long-term effects cannot be assessed. Moreover, although a wide array of control variables was used, some unmeasured factors could introduce residual confounding. Future research would benefit from a longitudinal approach to capture the temporal aspects of social interaction and its impact on MH, offering a more dynamic perspective on how these relationships may evolve and potentially strengthen or weaken over time.

### 5.4 Discussion on the impact of regional differences on policy implementation

The study’s findings suggest distinct regional differences in how social interaction affects mental health (MH) among older adults in the Greater Bay Area (GBA), with stronger positive effects observed in Hong Kong and Macao compared to Guangdong. In Hong Kong and Macao, extensive urban infrastructure and well-established community support systems provide older adults with diverse opportunities for organized activities, access to public recreational spaces, and formal social networks. These factors amplify the beneficial impact of social interactions on MH, as older adults can participate in various community and cultural activities that foster a sense of belonging and emotional well-being. Given these conditions, policies in Hong Kong and Macao could further enhance MH by expanding age-friendly community centers, promoting inclusive recreational programs, and increasing accessibility to social engagement platforms specifically tailored for older adults (70). In Guangdong, however, particularly in rural areas, limited access to social infrastructure (71) and organized activities may restrict the effectiveness of social interaction in promoting MH. Older adults in these regions often rely more heavily on family-based support networks, which can lead to over-reliance and potentially negative effects on autonomy and self-esteem. Therefore, policies aimed at improving MH in Guangdong should focus on establishing accessible social and recreational facilities, creating mobile community services, and implementing outreach programs that encourage independent social engagement. Such initiatives could include providing local community centers with multi-functional spaces, equipping mobile community service units with trained staff to visit remote areas, and offering regular social skill workshops designed to empower older adults to build and maintain independent networks of friends and peers. Additionally, introducing initiatives that reduce dependency on family support—such as community-led activities and peer support networks—may help older adults in rural Guangdong achieve better MH outcomes by fostering independence and broader social integration. These targeted approaches should be supported by local government funding and partnerships with non-governmental organizations to ensure sustainable operation and widespread access. This tailored, region-specific approach can bridge the urban–rural gap and ensure that older adults across the GBA benefit equitably from social interaction opportunities.

In comparison to international contexts, the findings of this study align with similar trends observed in other regions with diverse urban–rural divides. For instance, studies in Western countries such as the United States and Europe have found that urban environments, with their greater access to infrastructure and social services, tend to offer older adults more opportunities for social engagement (72), which correlates with better mental health outcomes. Conversely, rural areas in these countries, where social services are often limited, show similar challenges to those faced by older adults in rural Guangdong, including increased reliance on family support and a lack of formal social networks. In contrast, Scandinavian countries, which have strong social safety nets and well-integrated healthcare systems, provide a model for how both urban and rural populations can benefit from social support systems. These countries have implemented region-specific policies that offer community-based services tailored to the needs of older adults, including both urban and rural settings, to reduce social isolation and improve MH outcomes.

Furthermore, these findings have broader relevance to other urban–rural contexts where disparities in infrastructure and social resources may exist. Implementing region-specific approaches that address local social and economic conditions could help optimize MH outcomes in older populations. In urban settings, for example, increasing investment in public transit to ensure easy access to recreational facilities and developing digital platforms that connect older adults to community events and volunteer opportunities could further enhance social engagement and mental health. For urban settings, policies that support a variety of community programs and formalized social networks may yield substantial MH benefits. In rural areas, however, efforts to reduce dependence on family-based support systems and promote independent social engagement could mitigate potential negative impacts of over-reliance on family support. Rural-focused initiatives might include establishing regular community fairs or rotating cultural events in different villages to create consistent opportunities for older adults to socialize. These international comparisons suggest that integrating community-based solutions, enhancing social capital, and promoting intergenerational solidarity are critical components of effective policy in diverse global contexts. Consequently, the GBA serves as a model for how nuanced, region-sensitive policies can be adapted to improve MH outcomes in diverse settings.

## 6 Conclusions and implications

### 6.1 Conclusion

This study was based on the tracking survey in the GHMGBA, which included older adults aged 65 years and older. A comprehensive analysis of the effects of social interaction on older adults’ MH and the role of intergenerational support was conducted. This study found that regardless of the type of social interaction in which older adults participate in the GHMGBA, it significantly enhances their psychological health. However, older adults in the GHMGBA currently participate in social interaction at a relatively low rate. The three dimensions of intergenerational support – financial support, emotional support, and daily life support – mediated the relationship between social interaction and older adults MH. However, the mediating effects of different dimensions of intergenerational support were significantly different, with the mediating effects of economic support and emotional support being positive and daily life support being negative. In addition, this study makes some suggestions in order to improve older adults’ MH: (1) Governments may enhance support systems and create more opportunities for older adults to engage in social activities. (2) Families should strengthen intergenerational support for older adults, promote communication among family members, and increase the opportunities for the older adults to participate in social interaction. (3) It’s important to improve the older adults’ social support system. In order to provide them the chance to participate, more volunteer services and social interaction need to be offered.

### 6.2 Implications

#### 6.2.1 Policy implications

1. Policymakers should prioritize the development of community-based social engagement programs that provide older adults with more opportunities for meaningful social interaction. Community engagement has been shown to improve both mental and physical health, and structured activities can create spaces for older adults to build new social networks, thus reducing loneliness and enhancing their sense of belonging. In urban areas such as Hong Kong and Macao, where social infrastructure is more developed, enhancing access to community centers, parks, and organized social activities can further amplify the positive effects of social engagement. These programs could include age-friendly initiatives, such as cultural, recreational, and health-related activities designed specifically for older adults, enabling them to remain active participants in society. However, implementing such programs may face challenges such as funding constraints and space limitations in densely populated urban areas. Governments and policymakers must explore innovative funding mechanisms, such as public-private partnerships or philanthropic donations, to ensure sustainable program financing. Additionally, cultural resistance may arise, particularly in conservative communities, where older adults might be hesitant to participate in activities outside the family sphere. Policymakers should work to address these concerns through awareness campaigns that highlight the benefits of community engagement while respecting local values. Moreover, policymakers should consider implementing pilot programs that allow for gradual scaling. By starting with smaller-scale initiatives in select urban neighborhoods, it becomes possible to assess their effectiveness, identify potential barriers, and refine the programs before expanding citywide. This step-by-step approach ensures that community-based engagement initiatives are both practical and sustainable.
2. In more rural areas of Guangdong, targeted interventions are needed to improve access to social services and reduce older adults’ dependence on family support, which, when over-relied upon, has been shown to negatively affect mental health. Developing mobile health services, community outreach programs, and elder support networks in rural areas could help bridge the gap in service provision. Additionally, governments could promote intergenerational community programs that foster interaction between the younger and older generations, enhancing mutual support while allowing older adults to remain socially engaged without overburdening family members. However, rural areas may face logistical and infrastructural challenges, such as inadequate transportation networks, which limit older adults’ ability to access these services. Moreover, financial limitations may restrict the implementation and scalability of such interventions. Local governments must address these issues by allocating special budgets for rural development, improving transportation, and leveraging digital technology to reach older adults remotely. Cultural resistance may also present a challenge, as older adults in rural areas may prioritize family-based care over external social engagement. Tailored communication strategies that emphasize the compatibility of community programs with traditional family values can help mitigate this resistance. Another consideration is to integrate local community leaders into program development and execution. By involving trusted local figures—such as village elders or long-standing community volunteers—policymakers can build trust and increase program participation rates. This approach helps ensure that the programs are seen as locally relevant and culturally acceptable, reducing resistance and fostering long-term engagement.
3. Policy frameworks that incentivize local governments and NGOs to implement region-specific programs can address the urban–rural divide in resources. By integrating digital solutions, such as telehealth and online social platforms, governments can enhance older adults’ access to healthcare and social networks, particularly in areas where physical access to services is limited. Nevertheless, implementing digital solutions comes with its own challenges, such as the digital divide, where older adults in rural areas or lower-income groups may lack the necessary digital literacy or access to technology. Governments must invest in digital education programs and subsidize devices and internet access for older adults in underserved areas. Furthermore, the rapid pace of technological change may deter older adults from adopting new platforms. Continuous user-friendly design and ongoing support are essential to ensure that digital solutions are accessible and effective for this demographic. In addition to these investments, policymakers could establish regional technology hubs where older adults can receive hands-on support and training. These hubs would provide a central location for learning new digital skills, troubleshooting issues, and fostering peer-to-peer mentorship among older users. Over time, such centers could become an integral part of the digital inclusion strategy, empowering older adults to engage confidently with online social and healthcare platforms.

#### 6.2.2 Practical implications

1. For practitioners, it is important to tailor interventions to the specific needs of older adults in different regions. Programs that encourage participation in social activities, while balancing intergenerational support, should be developed to improve older adults’ mental health. For instance, group activities that foster social interaction, physical exercise, and mental stimulation could be organized in urban settings, while community-led initiatives that strengthen social bonds may be more appropriate for rural areas.
2. Training programs for caregivers and healthcare professionals should emphasize the importance of balancing necessary support and fostering independence among older adults. Caregivers should be trained to recognize when providing excessive daily life support could undermine an older adult’s autonomy and contribute to mental health decline. Emphasizing a person-centered care model that respects the individual’s need for autonomy while offering support when needed can lead to better outcomes.
3. Practitioners should implement monitoring and feedback mechanisms to assess the effectiveness of social programs and adjust them based on regional and demographic needs. Regular assessments of older adults’ mental and emotional health, combined with feedback from participants, can help refine programs to better meet the needs of various populations within the Greater Bay Area.
4. Promoting public-private partnerships can enhance resource allocation and the reach of social programs. By working with local businesses and civil society organizations, practitioners can mobilize additional resources, such as volunteer networks, financial support for low-income older adults, and technology access initiatives, to create more robust and sustainable programs that benefit the mental health of the older adult population across the region.

## 7 Limitations and recommendations for future research

Based on Social Exchange Theory, this study reveals the impact of social interaction and intergenerational support on the mental health (MH) of older adults through a survey conducted in the Greater Bay Area (GHMGBA). However, several limitations should be acknowledged. First, there is potential bias in data selection. This study relies on cross-sectional data, which limits our ability to assess the cumulative or long-term effects of social interaction and intergenerational support on older adults’ MH. Without longitudinal data, we cannot determine how changes in social interactions over time might influence MH, which may affect the interpretation of the results, particularly in understanding causal relationships. Additionally, the habits and social behaviors of older adults may evolve rapidly with ongoing societal changes, potentially creating timeliness issues and a lag between data collection and current realities. Furthermore, the cross-sectional design prevents us from controlling for unmeasured confounders, such as personality traits or baseline levels of social capital, which are known to influence both social interaction and mental health. These unmeasured factors may introduce bias into the observed relationships and limit the study’s ability to isolate causal effects. Potential self-selection bias is another limitation that needs consideration. Older adults who participate more frequently in social interactions may already possess unobserved characteristics—such as better physical health, higher levels of self-efficacy, or stronger pre-existing social networks—that differentiate them from less socially active individuals. This self-selection may skew the observed relationships and complicate interpretations of causality. Addressing self-selection bias in future research through techniques like instrumental variable analysis or matching methods would provide a more nuanced understanding of these relationships.

Second, potential bias in variable selection may also affect the results. While this study examines financial, emotional, and daily life support as mediating variables, it overlooks two-way intergenerational support in emotional terms, which might play a significant role in shaping MH outcomes. Similarly, the independent variable of social interaction lacks data on political social activities, such as voting or participation in elections, which may contribute to the social engagement and psychological well-being of older adults. Additionally, the study does not explicitly account for structural and environmental factors, such as neighborhood cohesion or access to community resources, which could influence the level and quality of social interaction. Neglecting these factors may lead to an incomplete understanding of the contextual determinants of mental health.

Finally, measurement issues should be considered. The study relies heavily on self-reported data, which may introduce reporting bias or social desirability bias. The lack of validated objective measures for certain constructs, such as the frequency and quality of social interactions, may limit the reliability of the findings. Incorporating more objective or mixed-method measures in future research would strengthen the validity of the results and provide a more robust foundation for understanding the complex relationships among social interaction, intergenerational support, and mental health.

To address these limitations, future research should incorporate longitudinal data to study the dynamic processes of social interaction, intergenerational support, and MH over time. Additionally, expanding the dimensions of the variables, including two-way emotional support and political social interaction, will help provide a more comprehensive understanding of the factors influencing older adults’ mental health. Future studies should also control for personality traits, social capital, and environmental factors, employing multilevel modeling or propensity score matching techniques to better address potential confounding effects and clarify the causal mechanisms underlying these relationships.

## Data Availability

The original contributions presented in the study are included in the article/supplementary material, further inquiries can be directed to the corresponding authors.
